# A Retrospective Review of Swallowing Outcomes for Oropharyngeal and Laryngeal Cancer Patients After Chemoradiation: A Single Institution Report

**DOI:** 10.7759/cureus.102501

**Published:** 2026-01-28

**Authors:** Austin J Halupnik, Shrikiriti S Rajan, Brian Peterson, Bradley Loeffler, Carryn Anderson

**Affiliations:** 1 Radiation Oncology, University of Iowa Carver College of Medicine, Iowa City, USA; 2 Radiation Oncology, University of Iowa Health Care, Iowa City, USA; 3 Otolaryngology, University of Iowa Health Care, Iowa City, USA; 4 Statistics, University of Iowa Health Care, Iowa City, USA

**Keywords:** chemoradiation, dysphagia, larygneal cancer, oropharyngeal cancer, radiation

## Abstract

Purpose: To collect our institutional experience and assess dysphagia prior to, during, and after chemoradiation for oropharyngeal and laryngeal cancer patients. This data can be used for future comparison with patients treated with newer radiation therapy techniques that are designed to decrease cumulative dose to swallowing structures.

Methods: A retrospective review of oropharyngeal and laryngeal cancer patients treated with chemoradiation with curative intent at the University of Iowa Healthcare (UIHC) from 2019-2022. Manual chart review identified baseline patient data, tumor characterization, and the following dysphagia measures: EAT-10, Penetration and Aspiration Scale (PAS), and oropharyngeal motility study (OPMS). Linear mixed effects regression was used to estimate the rate of change in mean scores from baseline, and to assess differences in baseline scores and the rate of change across disease and clinical characteristics.

Results: Of the 109 patients, 89 patients were treated for oropharyngeal cancer and 20 were treated for laryngeal cancer. There was a statistically significant increase in the mean EAT-10 scores from baseline to initial follow-up (10.14 vs. 13.27, p=0.03) for all patients. The rate of change in EAT-10 scores also significantly differed (p<0.01) based on whether the baseline EAT-10 assessment was prior, during, or after radiation therapy. Mean PAS scores were significantly greater at initial follow-up compared to baseline (3.94 vs. 3.14, p=0.04) for all patients. Mean baseline PAS scores significantly differed between larynx and oropharynx patients (4.63 vs. 2.81, p=0.01). There were no statistically significant differences between laryngeal and oropharyngeal patients in OPMS assessment of swallowing impairment. Patient-reported dysphagia via EAT-10 scores generally mirrored clinician assessments via PAS and OPMS.

Conclusions: Our analysis indicates that dysphagia worsens in oropharyngeal and laryngeal cancer patients in the months immediately following chemoradiation, with more self-perceived issues with swallowing and higher degrees of penetration and aspiration. Laryngeal cancer patients have higher degrees of penetration and aspiration than oropharyngeal cancer patients at baseline. Future prospective studies are warranted to evaluate swallowing outcomes in patients treated with newer radiation techniques.

## Introduction

Head and neck cancers (HNC) are among the most common global malignancies, with high mortality [1]. Oropharyngeal and laryngeal cancers comprise a large portion of current HNC [2], and individuals with oropharyngeal cancers are currently presenting at younger ages due to an increase in human papillomavirus (HPV)-positive oropharyngeal cancer [3]. The introduction of concurrent chemoradiation has improved the survival of those with HNC, particularly those with HPV-positive oropharyngeal cancer [4]. Although the utilization of chemoradiation has led to increased survival among those with HNC, side effects include dysphagia, mucositis, pain, edema of soft tissues, xerostomia, muscle atrophy, and loss of sensation [5]. 

The most common long-term side effect of chemoradiation is dysphagia [6]. This ranges from difficulty swallowing solid foods to a dependency on percutaneous endoscopic gastrostomy. An estimated 39% to 63% of those who undergo radiotherapy for HNC develop dysphagia within two years after treatment, with varied severity [7]. Decreased swallowing function may be the result of edema, neuropathy, or fibrosis from chemoradiation, which can lead to damage to the pharyngeal and laryngeal musculature involved in swallowing [8]. 

Dysphagia predisposes individuals to morbidity after treatment, including pneumonia, prolonged use of a feeding tube, and weight loss [9]. Patients with dysphagia are also at an increased risk for aspiration of food and liquids, which is dangerous for the many HNC patients who do not cough in response to aspiration [10]. Dysphagia also broadly affects patient quality of life (QOL), as individuals may require changes in eating patterns and experience pain and discomfort. In addition to these physical manifestations, patients may experience psychological distress due to a loss of their emotional and social connections to eating [11]. Given the consequences of dysphagia, it is important to detect dysphagia promptly following chemoradiation to allow for swallowing rehabilitation and nutritional assistance [12]. 

By monitoring for and managing dysphagia in follow-up, fewer complications of dysphagia may materialize [13]. There are currently an assortment of different measures for dysphagia, including QOL questionnaires, imaging techniques, and methods that look at other aspects such as diet and utilization of feeding tubes [14]. The EAT-10 is a self-administered dysphagia assessment with excellent internal consistency, reproducibility, and validity [15]. The oropharyngeal motility study (OPMS), often referred to as videofluoroscopic swallowing study (VFSS), is the current gold-standard swallowing evaluation, allowing real-time observation of bolus and anatomical structures [16]. The Penetration and Aspiration Scale (PAS) is assessed from video of OPMS and has an excellent sensitivity for both penetration and aspiration of the airway [17]. 

In this study, we aimed to evaluate three prominent dysphagia metrics, OPMS evaluation, PAS, and patient-reported EAT-10, to analyze functional evidence of dysphagia as well as patient perception of dysphagia prior to, during, and following definitive chemoradiation in oropharyngeal and laryngeal HNC patients. This data will serve as a baseline characterization of dysphagia in oropharyngeal and laryngeal cancer patients treated with volumetric modulated arc therapy (VMAT) at our institution, for future comparison against radiation-sparing techniques. Additionally, there is descriptive analysis comparing oropharyngeal patients versus laryngeal patients, which is not currently available in our review of the literature.

This article was previously presented as a meeting abstract at the ACRO 2024 Summit on March 16, 2024. 

## Materials and methods

Patient cohort

In this institutional review board-approved study (IRB #201701826), we conducted a retrospective, single-institution cohort study of patients with biopsy-proven oropharyngeal or laryngeal cancer. Eligibility criteria included patients treated with chemoradiation with curative intent for either oropharyngeal or laryngeal cancer who received treatment at the University of Iowa Healthcare (UIHC) from 2019-2022. Exclusion criteria included failure to complete radiation treatment, failure to attend two speech therapy visits, death during treatment, and concurrent non-oropharyngeal or laryngeal neoplasm. The study cohort comprised all eligible patients meeting these criteria during the study period.

Treatment and follow-up

The initial dose of radiation was given between 2019 and 2022. Patients received standard-of-care radiation with curative intent with VMAT. All but one patient received concurrent chemotherapy. Monochemotherapy regimens included high (100 mg/m2) and low (40 mg/m2) dose cisplatin. Combination chemotherapies included carboplatin/taxol, cisplatin/paclitaxel, carboplatin/etoposide, carboplatin/5FU, and cisplatin/etoposide. Follow-up visits generally occurred at four weeks post-radiation therapy and at three-to-six-month intervals over the following five years. Patients had the opportunity to visit with speech therapy at each of these visits, but follow-up with speech therapy was heterogeneous. Speech therapy visits were conducted by one of three speech-language pathologists (SLPs) at UIHC using standardized protocols. Variation in follow-up frequency may introduce bias, as patients seen more frequently may have more complete outcome data. Linear mixed-effects models were used to account for unequal follow-up and partially mitigate this bias.

Demographics and clinical variables

Demographic and clinical information was obtained on patients, including age at diagnosis, sex, smoking history, smokeless tobacco history, diabetic status, HPV histology, staging (American Joint Committee on Cancer, eighth edition), tumor subsite, and use of feeding tubes. All demographic information was collected through chart review at first radiation oncology or medical oncology visit. Cancer subsite, HPV status, and staging were obtained through chart review from radiation oncology visits prior to chemoradiation. Feeding tube use was determined by chart review of procedures prior to, during, and following treatment, and included percutaneous endoscopic gastrostomy (PEG), nasogastric (NG), and nasojejunal (NJ) tubes.

Outcomes

Speech therapy visits were reviewed for multiple outcomes documented by SLPs. Patient-perceived dysphagia was measured with the EAT-10 scale, while the OPMS and PAS assessed dysphagia using fluoroscopic imaging.

The EAT-10 scale (scaled 0-40: 0 = no swallowing problems, 40 = severe dysphagia) is a patient-reported symptom-specific outcome tool that was used to assess the patients’ self-perceived issues with dysphagia. A score of 3 or higher is indicative of swallowing dysfunction [15]. EAT-10 was evaluated heterogeneously among patients in the periods prior to, during, and after radiation.

Impairment of swallowing phases and the PAS were acquired from OPMS, during which patients swallow various materials mixed or coated in contrast with concurrent imaging. SLPs grade both impairment of swallowing and the PAS using standardized observations. Impairment of swallowing phases from OPMS (scaled from normal to severe impairment) was done for the four phases of swallowing (oral preparatory, oral, pharyngeal, and cervical/esophageal) and used to assess dysphagia by anatomical region [16]. The PAS (scaled 1-8: 1 = no penetration, 8 = aspiration with no attempt to clear) assessed the degree of penetration or aspiration of swallowed materials into the airway. A PAS score of 3-5 demonstrates penetration outside of normal limits, while a score of 6-8 demonstrates aspiration [17].

Statistical analysis

Linear mixed effects regression was used to estimate the rate of change in mean swallowing assessment scores from baseline, and to assess differences in baseline scores and the rate of change across disease and clinical characteristics. Random effects were included to account for the longitudinally correlated nature of repeated swallowing assessments at unequal follow-up with a spatial power correlation structure. The effect of follow-up visit was evaluated categorically, and assessments of disease and clinical characteristics were done holistically via the interaction between follow-up visit and the covariate of interest. Specifically, visit-specific means were estimated from each model and the global effect of time was evaluated via an F-test. In swallowing assessments with more than two time points (EAT-10), all pairwise differences between visits were subsequently assessed via t-tests. All statistical testing was two-sided and assessed for significance at the 5% level using SAS v9.4 (SAS Institute, Cary, NC, USA). 

## Results

Baseline patient demographics and disease characteristics are summarized in Table 1. Patients were separated by primary tumor site into laryngeal cancer (18%, n=20) and oropharyngeal cancer (82%, n=89) cohorts. Median age at diagnosis for all patients was 63 years (range, 24-86). Eighty-nine percent of patients were male (n=97) and 58% had a prior smoking history (n=63). Seventeen percent of patients had a prior smokeless tobacco history (n=18). Ninety percent of patients with oropharyngeal cancer had HPV positive disease (n=78). Laryngeal cancer patients were most commonly overall stage 3 (40%, n=8), followed by stage 4a (35%, n=7), and stage 4b (25%, n=5). Oropharyngeal cancer patients were most commonly overall stage 1 (43%, n=38), followed by stage 2 (29%, n=26), stage 3 (19%, n=17), stage 4a (8%, n=7), and stage 4b (1%, n=1). 

**Table 1:** Patient demographics and disease characterization. HPV: human papillomavirus; AJCC v8 T stage: American Joint Committee on Cancer, eighth edition, T stage; RT: radiation therapy. Age at diagnosis measured in years.

Covariate	Statistics	Level	Larynx N=20	Oropharynx N=89	Total N=109
Sex	N (%)	Male	18 (90.0)	79 (88.8)	97 (89.0)
	N (%)	Female	2 (10.0)	10 (11.2)	12 (11.0)
Subsite	N (%)	Tonsil	0 (0)	42 (47.2)	42 (38.5)
	N (%)	Base of Tongue	0 (0)	42 (47.2)	42 (38.5)
	N (%)	Oropharynx Other	0 (0)	5 (5.6)	5 (4.6)
	N (%)	Supraglottis	11 (55.0)	0 (0)	11 (10.1)
	N (%)	Glottis	4 (20.0)	0 (0)	4 (3.7)
	N (%)	Subglottis	1 (5.0)	0 (0)	1 (0.9)
	N (%)	Larynx Other	4 (20.0)	0 (0)	4 (3.7)
HPV Status	N (%)	Positive	0 (0)	78 (89.7)	78 (72.9)
	N (%)	Negative	20 (100)	9 (10.3)	29 (27.1)
		Missing	0	2	2
AJCC v8 T Stage	N (%)	1	0 (0)	24 (27.0)	24 (22.0)
	N (%)	2	2 (10.0)	28 (31.5)	30 (27.5)
	N (%)	3	11 (55.0)	21 (23.6)	32 (29.4)
	N (%)	4	0 (0)	14 (15.7)	14 (12.8)
	N (%)	4a	7 (35.0)	2 (2.2)	9 (8.3)
Overall Stage	N (%)	1	0 (0)	38 (42.7)	38 (34.9)
	N (%)	2	0 (0)	26 (29.2)	26 (23.9)
	N (%)	3	8 (40.0)	17 (19.1)	25 (22.9)
	N (%)	4a	7 (35.0)	7 (7.9)	14 (12.8)
	N (%)	4b	5 (25.0)	1 (1.1)	6 (5.5)
Smoking Status	N (%)	Former Smoker	12 (60.0)	32 (36.0)	44 (40.4)
	N (%)	Current Smoker	6 (30.0)	13 (14.6)	19 (17.4)
	N (%)	Never Smoker	2 (10.0)	44 (49.4)	46 (42.2)
Smokeless Tobacco	N (%)	Former User	3 (15.0)	11 (12.4)	14 (12.8)
	N (%)	Current User	1 (5.0)	3 (3.4)	4 (3.7)
	N (%)	Never Used	16 (80.0)	75 (84.3)	91 (83.5)
Diabetes	N (%)	No	17 (85.0)	78 (87.6)	95 (87.2)
	N (%)	Yes	3 (15.0)	11 (12.4)	14 (12.8)
Was Feeding Tube Placed During Radiation?	N (%)	Yes	8 (40.0)	44 (49.4)	52 (47.7)
	N (%)	No	9 (45.0)	33 (37.1)	42 (38.5)
	N (%)	Feeding tube dependent due to tumor prior to treatment	3 (15.0)	9 (10.1)	12 (11.0)
	N (%)	Feeding tube dependent after treatment	0 (0)	3 (3.4)	3 (2.8)
Age at Diagnosis	N		20	89	109
	Mean		62.7	61.3	61.6
	Median		65.0	62.0	63.0

**Figure 1:**
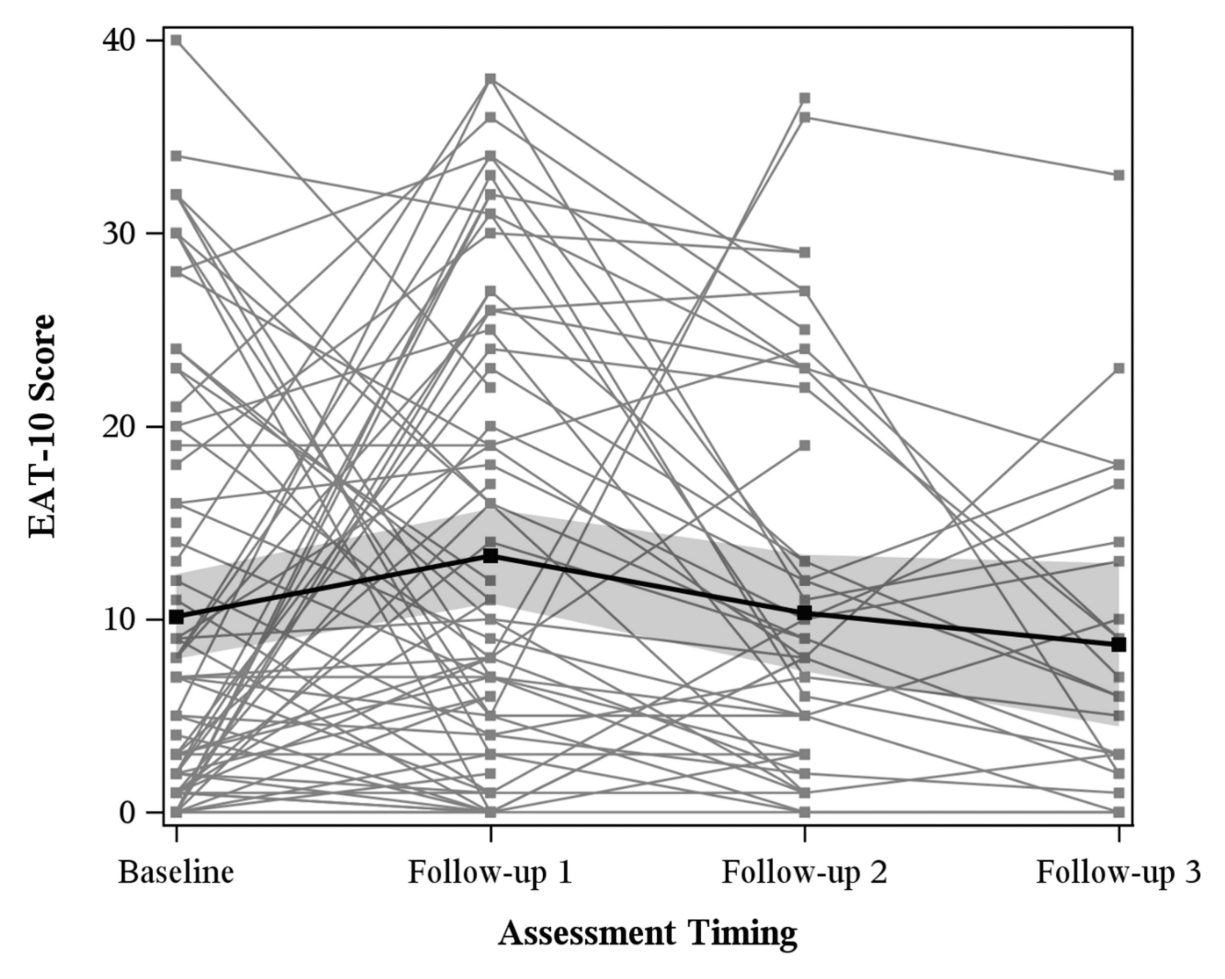
EAT-10 scores at baseline, follow-up 1, follow-up 2, and follow-up 3 for all patients, with the global mean estimated at each timepoint. EAT-10 was patient-reported and on a scale of 0 (no swallowing problems) to 40 (severe swallowing problems). The mean EAT-10 score was found to significantly vary across time (p=0.04). Specifically, there was a statistically significant difference (p=0.03) between mean baseline EAT-10 (10.14) and follow-up 1 (13.27). EAT-10 was used with permission from its copyright holder [15].

EAT-10 results for all patients are summarized in Figure 1. Median timing of the baseline EAT-10 was 1.4 months prior to initiation of radiation therapy, 1.1 months after initiation of radiation therapy for follow-up 1, 3.2 months after initiation of radiation therapy for follow-up 2, and 6.3 months after initiation of radiation therapy for follow-up 3. The mean EAT-10 score was found to significantly vary across time (p=0.04). Specifically, there was a statistically significant difference (p=0.03) between mean baseline EAT-10 (10.14) and follow-up 1 (13.27). There was no statistically significant difference between any other intervals. The mean EAT-10 at follow-up 2 was 10.31, while the mean EAT-10 at follow-up 3 was 8.67. 

**Figure 2:**
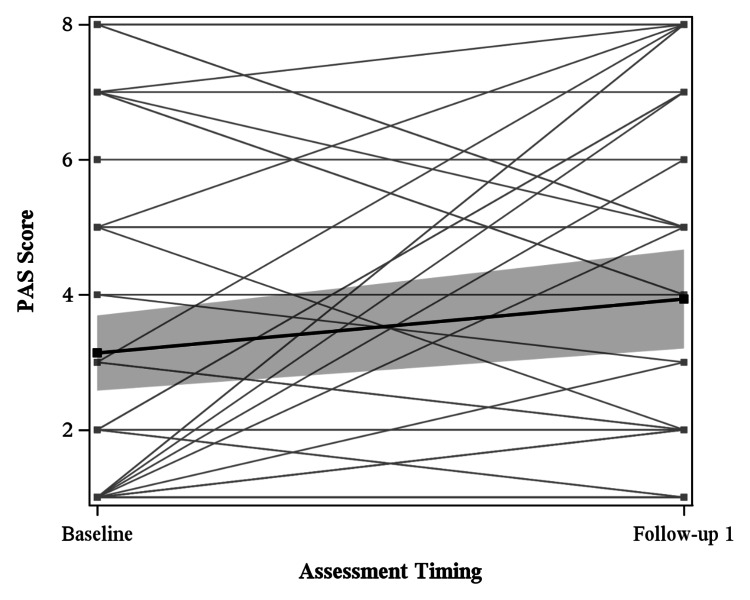
Penetration and Aspiration Scale (PAS) results for all patients at baseline and follow-up 1, with the global mean estimated at each timepoint. PAS scores were evaluated by clinicians using the 1 (no penetration) to 8 (aspiration with no attempt to clear) scale following visualization of swallowing. There was a statistically significant (p=0.04) difference between mean baseline PAS (3.14) and follow-up 1 (3.94).

PAS results for all patients are summarized in Figure 2. PAS data was collected during OPMS. Median timing for baseline PAS for all patients was 1.5 months prior to treatment with radiation therapy, while median timing for follow-up 1 was 3.5 months after the start of treatment with radiation therapy. The difference between mean baseline PAS (3.14) and follow-up 1 (3.94) was statistically significant (p=0.04).

**Figure 3:**
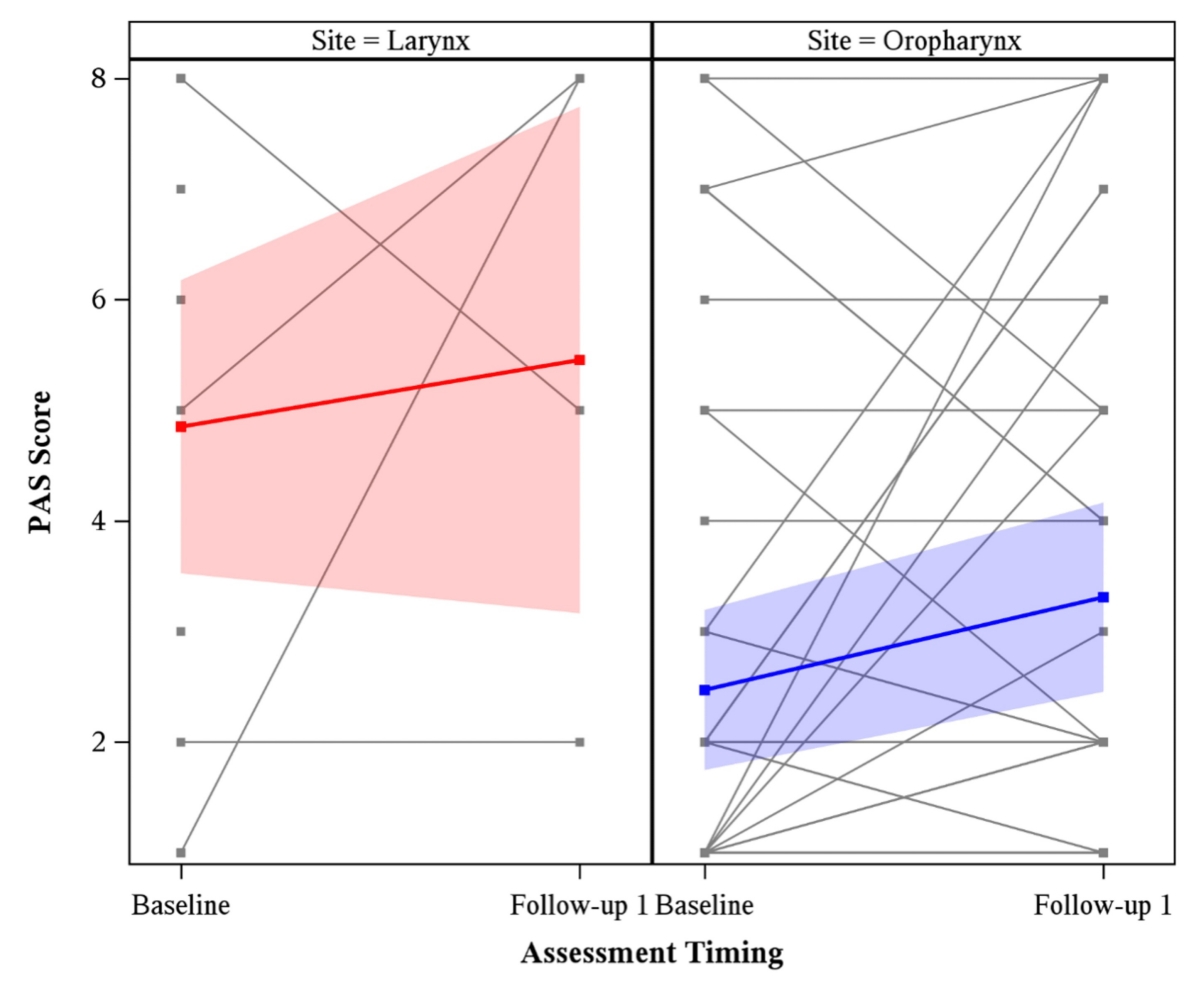
Penetration and Aspiration Scale (PAS) scores for larynx vs. oropharynx patients at baseline and follow-up 1, with the global mean estimated at each timepoint. Mean baseline PAS for laryngeal cancer patients (4.63) was statistically significantly higher (p=0.04) than mean baseline PAS for oropharyngeal cancer patients (2.81).

PAS results are separated by laryngeal and oropharyngeal cohorts in Figure 3. Mean baseline PAS for laryngeal cancer patients (4.63) was statistically significantly higher (p=0.04) than mean baseline PAS for oropharyngeal cancer patients (2.81). Mean follow-up 1 PAS for laryngeal cancer patients was 4.3 months, while mean follow-up 1 PAS for oropharyngeal cancer patients was 6.0 months.

**Figure 4:**
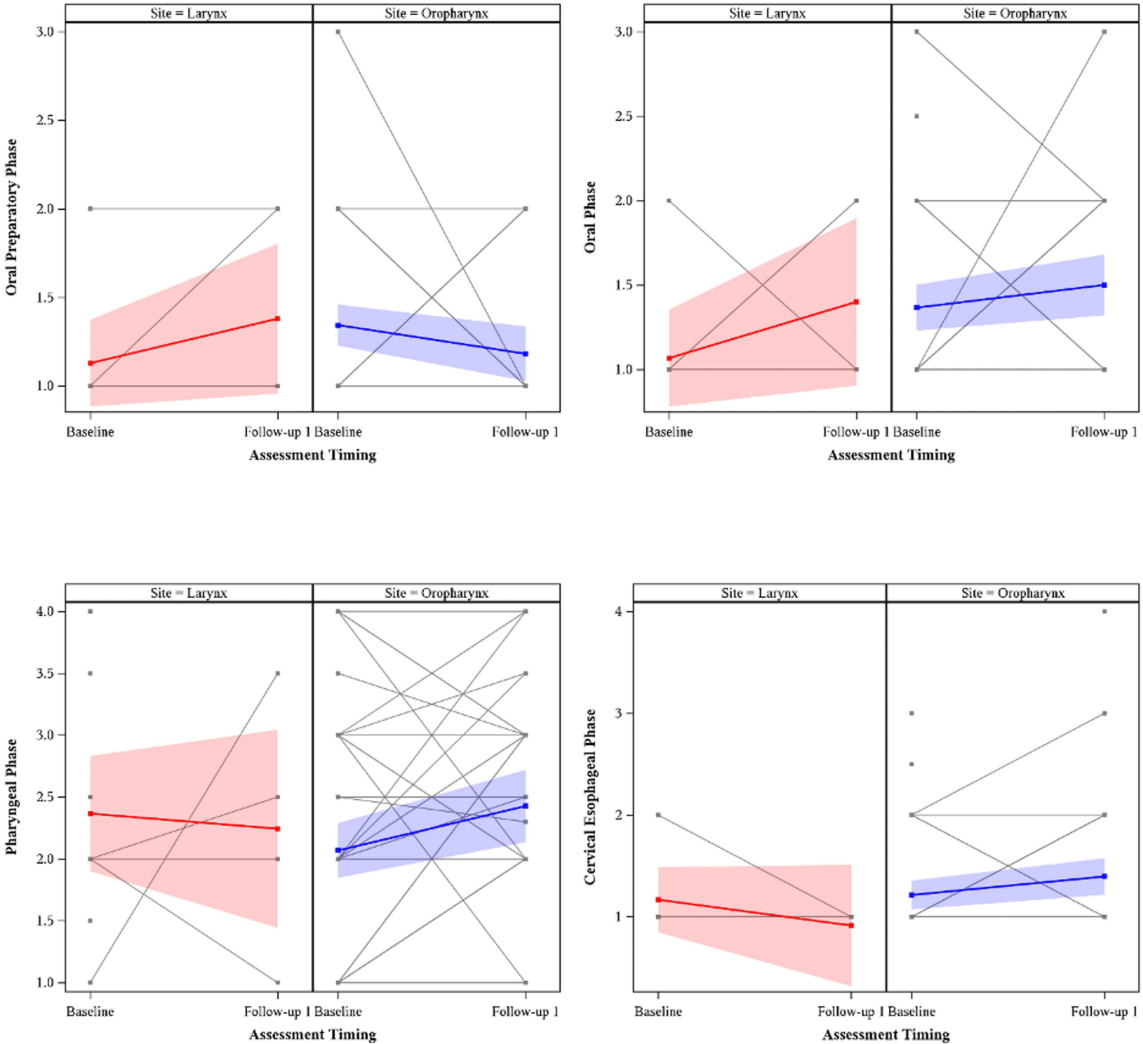
Oropharyngeal motility study (OPMS) results for larynx and oropharynx patients at baseline and follow-up 1, with the global mean estimated at each timepoint. Four phases of swallowing were evaluated by clinicians, with impairment rated from 1 (no impairment) to 4 (severe impairment) following visualization of swallowing. There were no statistically significant differences between baseline and follow-up 1 for either cohort in any swallowing phase, nor between baselines for the oropharyngeal and laryngeal groups for any swallowing phase.

Swallowing impairment results from OPMS are summarized in Figure 4. Laryngeal and oropharyngeal patients were separated by cohort for assessment of each phase of swallowing. OPMS indications included surveillance and worsening dysphagia, but were not standardized among patients. Median timing for baseline OPMS for all patients was 1.5 months prior to treatment with radiation therapy, while median timing for follow-up 1 was 3.5 months after treatment with radiation therapy. There were no statistically significant differences between baseline and follow-up 1 for either cohort in any swallowing phase, nor between baselines for the oropharyngeal and laryngeal groups for any swallowing phase.

**Figure 5:**
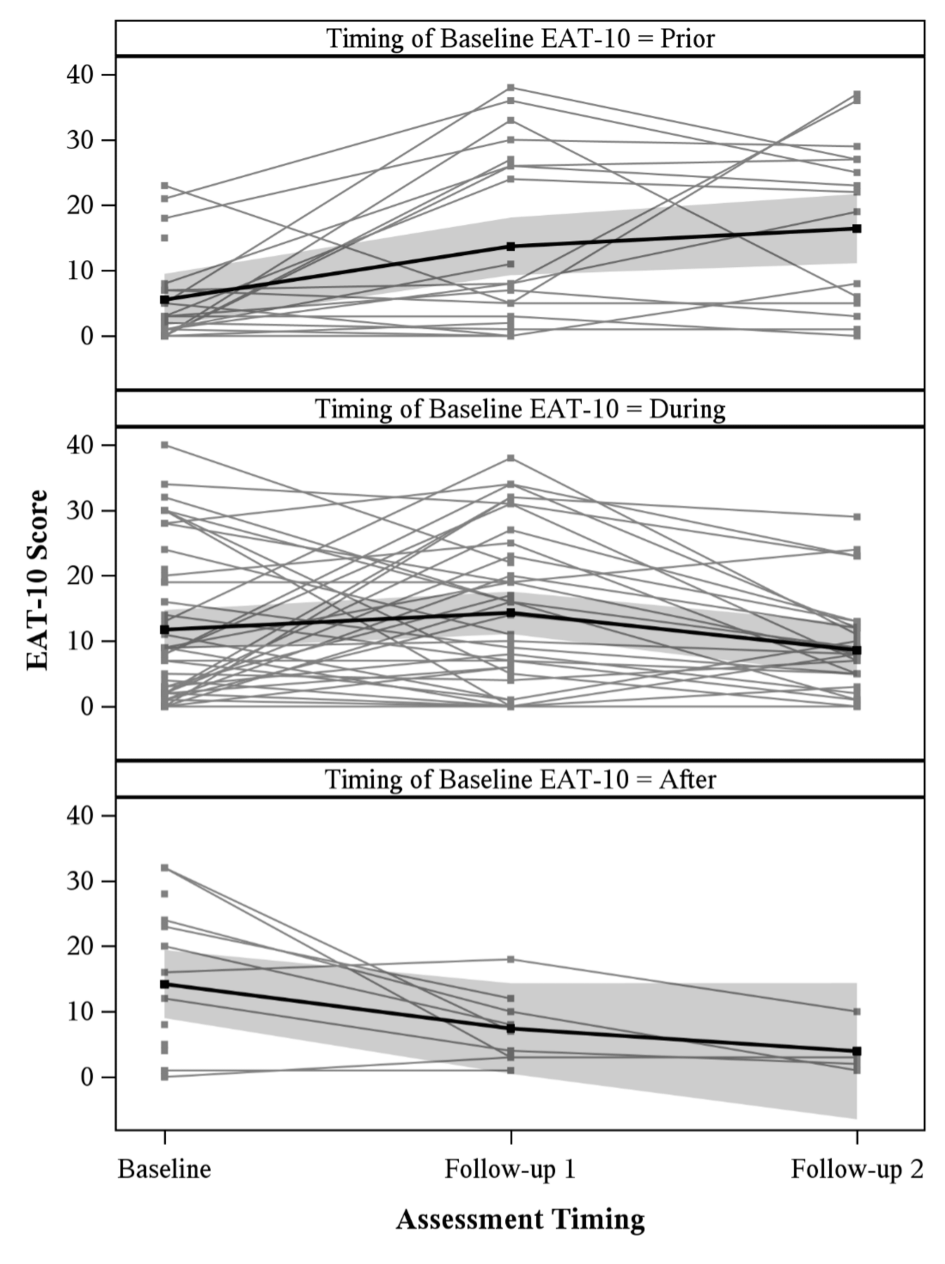
EAT-10 scores for all patients stratified by assessment timing in relation to radiation treatment. There was a statistically significant difference (p<0.01) in the rate of change in EAT-10 scores across assessment timing. EAT-10 was used with permission from its copyright holder [15].

EAT-10 results of all patients are stratified by the timing of baseline EAT-10 completion in Figure 5. Patients are separated by the timing of their baseline EAT-10 in reference to their initiation of radiation therapy: patients with baseline EAT-10 prior to start of radiation, during radiation, and after completion of radiation. There was a statistically significant difference (p<0.01) in the rate of change in EAT-10 scores between these three groups. Similar analysis of baseline scores in PAS and all swallowing phases of OPMS showed no significant differences based on timing of baseline assessment.

## Discussion

Of the 109 patients included in analysis, 89 with oropharyngeal cancer and 20 with laryngeal cancer, self-perceived dysphagia was assessed with EAT-10 and imaging-based dysphagia was assessed with PAS and OPMS swallowing impairment. Patients had significant dysphagia at baseline, affecting both their self-perceived ability to swallow and their observed ability to swallow, as seen in Figures 1, 2. The mean EAT-10 score of 10.14 indicated marked dysphagia at baseline. Analysis of EAT-10 baseline scores only prior to onset of radiation therapy (Figure 5) still showed abnormal EAT-10 baseline scores, evidence that elevated baseline EAT-10 scores were not solely due to heterogeneity of timing. Mean PAS scores were similarly elevated at baseline, with a mean of 3.15 outside of normal limits, indicating imaging-based swallowing impairment at baseline. Other studies have similarly displayed that tumor burden itself can cause dysphagia, leading to both abnormal EAT-10 scores [18] and penetration/aspiration [19] prior to treatment.

The degree of dysphagia was worst in the months immediately following chemoradiation. During this period, patients experienced more self-perceived issues with swallowing (EAT-10) and had higher degrees of penetration and aspiration (PAS). EAT-10 scores were highest at follow-up 1, a statistically significant increase from baseline, with scores generally trending downward at subsequent follow-ups (Figure 1). Median follow-up 1 timing was approximately one month after radiation completion, while median follow-up 2 was roughly three months after radiation completion, showing marked worsening of symptoms directly after radiation with improvement by three months. PAS scores were significantly worse at follow-up 1 in our analysis, 3.5 months after initiation of chemoradiation (Figure 2). The timing of this peak radiation toxicity is consistent with prior studies using both EAT-10 and PAS [12,20]. Although our analysis of PAS was truncated after follow-up 1 due to small sample size, further investigation is merited as others have reported chronic worsening of PAS beyond two years [21].

The laryngeal cancer cohort had a significantly higher degree of baseline penetration and aspiration than the oropharyngeal cohort, as seen in Figure 3. This is consistent with prior literature describing higher baseline PAS scores in patients with advanced T stage tumors in the larynx than early-stage laryngeal tumors or oropharyngeal tumors [22]. Heightened penetration and aspiration of laryngeal cancer patients prior to treatment may account for this observed impairment of swallowing [23]. Though analysis of follow-up 1 was limited, there was a larger increase in interval PAS score for laryngeal patients (1.4) than oropharyngeal patients (1.0), likely secondary to increased mean pharyngeal dose and laryngeal structural dysfunction at follow-up 1 with definitive treatment to a laryngeal primary compared to an oropharyngeal primary.

Patient-reported dysphagia via EAT-10 scores generally mirrors clinician assessments via PAS and OPMS. Both EAT-10 and PAS for all patients showed baseline swallowing deficits and worsening dysphagia from baseline to follow-up 1. Analysis of PAS was truncated and therefore did not allow for comparison of EAT-10 and PAS trends after follow-up 1. Previous literature has shown moderate association between EAT-10 and PAS, though this association was limited to one year after radiation [24,25].

There were no significant findings in analysis of OPMS data, as seen in Figure 4. However, there was a general increase in pharyngeal phase impairment in OPMS from baseline to follow-up 1 that coincided with the increase in EAT-10 from baseline to follow-up 1. Other recent studies have noted that the pharyngeal phase of swallowing has the strongest relationship with self-reported swallowing symptoms [25,26]. This further supports the notion that patient-perceived and imaging-based outcomes were consistent over the acute radiation toxicity phase.

Consistent timing of baseline and follow-up assessment is paramount in future prospective trials. This is particularly relevant for EAT-10, which varied significantly based on the timing of baseline assessment with reference to radiation treatment, as seen in Figure 5. This may suggest that EAT-10 is more influenced by acute toxicities of radiation than the imaging-based PAS and OPMS, consistent with prior literature suggesting that aspiration is a chronic manifestation of radiation toxicity [26]. Timing of baseline assessment of PAS and OPMS did not cause the rate of change in scores to significantly differ.

The unique value added in this small retrospective study is the descriptive comparison of laryngeal patients versus oropharyngeal patients as assessed with these three dysphagia assessments (EAT-10, PAS, OPMS) over time. Most prospective clinical trials testing chemoradiation in HNC that have analyzed dysphagia have analyzed all head and neck subsites together. They utilize patient-reported outcomes such as the MD Anderson Dysphagia Inventory (MDADI), the Functional Assessment of Cancer Therapy-Head and Neck (FACT-HN), or other validated questionnaires, but exclude objective functional measurements such as OPMS. MD Anderson has recently published their prospective longitudinal outcomes study of oropharyngeal cancer patients, inclusive of both functional clinician-reported and patient-reported outcomes performed at consistent timepoints [27].

Study limitations are secondary to its retrospective nature and heterogeneity of speech therapy. Follow-up with speech therapy varied, and timing of baseline assessments varied with respect to initiation of chemoradiation. Truncated follow-up with speech therapy in many patients resulted in limited longitudinal analysis of dysphagia. The small size of the laryngeal cohort limited analysis between cohorts. Our cohort is too small to be able to assess swallowing outcomes by primary tumor stage (T1-T2 vs T3-T4) and the laryngeal cohort is so small, confidence in discerning trends in comparison to oropharyngeal patients is limited. However, we hope our hypothesis-generating descriptive analysis inspires others to further analyze prospective clinical trial data by primary tumor subsite. For example, RTOG 1016 included both larynx and oropharyngeal patients and participants completed the EORTC Quality of Life Questionnaire-Head & Neck 35 at specified timepoints [28].

Conventional radiotherapy treatments currently in use, such as VMAT, allow for uniform distribution of radiation [29]. MR-guided radiation therapy is a relatively new technique that allows for increased accuracy in targeting radiation in soft tissues with similar to improved outcomes [30]. MR-Linac is an MR-guided radiation therapy that combines MRI with a linear accelerator. Unlike conventional radiotherapy, in which original plans for daily treatments remain the same for multiple weeks, MR-Linac technology includes the ability to adapt the radiation target and plan with each fraction, which may decrease cumulative radiation to adjacent organs at risk [31].

There is evidence that MR-guided radiation therapy may be able to reduce dysphagia in HNC patients. Mohamed et al. [32] utilized MRI to revise intensity-modulated radiation therapy (IMRT) plans every two weeks for oropharyngeal cancer patients. With the adaptive plans, the majority of organs at risk received a decreased radiation dose. There was a decrease in incidence of moderate-severe dysphagia and use of feeding tube six months post-radiation of 11% and 4%, respectively. Future prospective studies will compare the dysphagia for oropharyngeal and laryngeal cancer patients treated using MR-Linac to the dysphagia characterized in this study.

## Conclusions

This review of swallowing outcomes at our institution follows patterns previously published by others, indicating that dysphagia worsens in oropharyngeal and laryngeal cancer patients in the months immediately following chemoradiation. During this period, patients experience more self-perceived issues with swallowing and have higher degrees of penetration and aspiration. Patients treated for laryngeal cancer have higher degrees of penetration and aspiration than oropharyngeal cancer patients at baseline. Patient-reported dysphagia and imaging-based dysphagia generally mirrored one another in the acute toxicity period. Consistent timing of assessments is important for future studies, particularly for assessment of EAT-10. Future prospective studies will continue to evaluate swallowing outcomes at our institution for oropharynx and larynx cancer patients treated with newer radiation techniques.

## References

[1] Pezzuto F, Buonaguro L, Caponigro F (2015). Update on head and neck cancer: current knowledge on epidemiology, risk factors, molecular features and novel therapies. Oncology.

[2] Simard EP, Torre LA, Jemal A (2014). International trends in head and neck cancer incidence rates: differences by country, sex and anatomic site. Oral Oncol.

[3] Baijens LW, Walshe M, Aaltonen LM (2021). European white paper: oropharyngeal dysphagia in head and neck cancer. Eur Arch Otorhinolaryngol.

[4] Chow LQ (2020). Head and neck cancer. N Engl J Med.

[5] Ohba S, Yokoyama J, Kojima M, Fujimaki M, Anzai T, Komatsu H, Ikeda K (2016). Significant preservation of swallowing function in chemoradiotherapy for advanced head and neck cancer by prophylactic swallowing exercise. Head Neck.

[6] Wilson JA, Carding PN, Patterson JM (2011). Dysphagia after nonsurgical head and neck cancer treatment: patients' perspectives. Otolaryngol Head Neck Surg.

[7] Shinn EH, Jensen K, McLaughlin J, Garden AS, Fellman BM, Liang L, Peterson SK (2019). Interactive website for head and neck cancer patients: adherence and coping program to prevent dysphagia after radiation. Internet Interv.

[8] Hutcheson KA, Bhayani MK, Beadle BM, Gold KA, Shinn EH, Lai SY, Lewin J (2013). Eat and exercise during radiotherapy or chemoradiotherapy for pharyngeal cancers: use it or lose it. JAMA Otolaryngol Head Neck Surg.

[9] Semenov YR, Starmer HM, Gourin CG (2012). The effect of pneumonia on short-term outcomes and cost of care after head and neck cancer surgery. Laryngoscope.

[10] Nguyen NP, Frank C, Moltz CC (2006). Aspiration rate following chemoradiation for head and neck cancer: an underreported occurrence. Radiother Oncol.

[11] McQuestion M, Fitch M, Howell D (2011). The changed meaning of food: physical, social and emotional loss for patients having received radiation treatment for head and neck cancer. Eur J Oncol Nurs.

[12] Xinou E, Chryssogonidis I, Kalogera-Fountzila A, Panagiotopoulou-Mpoukla D, Printza A (2018). Longitudinal evaluation of swallowing with videofluoroscopy in patients with locally advanced head and neck cancer after chemoradiation. Dysphagia.

[13] Kraaijenga SA, Molen LV, Stuiver MM, Takes RP, Al-Mamgani A, Brekel MW, Hilgers FJ (2017). Efficacy of a novel swallowing exercise program for chronic dysphagia in long-term head and neck cancer survivors. Head Neck.

[14] Pedersen A, Wilson J, McColl E, Carding P, Patterson J (2016). Swallowing outcome measures in head and neck cancer--how do they compare?. Oral Oncol.

[15] Belafsky PC, Mouadeb DA, Rees CJ, Pryor JC, Postma GN, Allen J, Leonard RJ (2008). Validity and reliability of the Eating Assessment Tool (EAT-10). Ann Otol Rhinol Laryngol.

[16] Martin-Harris B, Jones B (2008). The videofluorographic swallowing study. Phys Med Rehabil Clin N Am.

[17] Rosenbek JC, Robbins JA, Roecker EB, Coyle JL, Wood JL (1996). A penetration-aspiration scale. Dysphagia.

[18] Leão I, Garcia C, Antunes P (2022). Acute impact of cancer treatment on head and neck cancer patients: FIT4TREATMENT. Cancers (Basel).

[19] Gawryszuk A, Bijl HP, van der Schaaf A (2021). Relationship between videofluoroscopic and subjective (physician- and patient- rated) assessment of late swallowing dysfunction after (chemo) radiation: results of a prospective observational study. Radiother Oncol.

[20] Lazarus CL, Husaini H, Hu K (2014). Functional outcomes and quality of life after chemoradiotherapy: baseline and 3 and 6 months post-treatment. Dysphagia.

[21] van der Laan HP, Van den Bosch L, Schuit E, Steenbakkers RJ, van der Schaaf A, Langendijk JA (2021). Impact of radiation-induced toxicities on quality of life of patients treated for head and neck cancer. Radiother Oncol.

[22] Starmer H, Gourin C, Lua LL, Burkhead L (2011). Pretreatment swallowing assessment in head and neck cancer patients. Laryngoscope.

[23] Shune SE, Karnell LH, Karnell MP, Van Daele DJ, Funk GF (2012). Association between severity of dysphagia and survival in patients with head and neck cancer. Head Neck.

[24] Bartlett RS, Kenz MK, Wayment HA, Thibeault SL (2022). Correlation between EAT-10 and aspiration risk differs by dysphagia etiology. Dysphagia.

[25] Arrese LC, Carrau R, Plowman EK (2017). Relationship between the Eating Assessment Tool-10 and objective clinical ratings of swallowing function in individuals with head and neck cancer. Dysphagia.

[26] Pauloski BR, Rademaker AW, Logemann JA (2002). Swallow function and perception of dysphagia in patients with head and neck cancer. Head Neck.

[27] Moreno A, Sahli AJ, Johnson F (2025). Stiefel MD Anderson OroPharynx cancer (MDA-OPC) cohort: a single-institution, prospective longitudinal outcomes study. BMJ Open.

[28] Gillison ML, Trotti AM, Harris J (2019). Radiotherapy plus cetuximab or cisplatin in human papillomavirus-positive oropharyngeal cancer (NRG Oncology RTOG 1016): a randomised, multicentre, non-inferiority trial. Lancet.

[29] Liu X, Li Z, Yin Y (2023). Clinical application of MR-Linac in tumor radiotherapy: a systematic review. Radiat Oncol.

[30] Henke L, Kashani R, Robinson C (2018). Phase I trial of stereotactic MR-guided online adaptive radiation therapy (SMART) for the treatment of oligometastatic or unresectable primary malignancies of the abdomen. Radiother Oncol.

[31] Randall JW, Rammohan N, Das IJ, Yadav P (2022). Towards accurate and precise image-guided radiotherapy: clinical applications of the MR-Linac. J Clin Med.

[32] Mohamed AS, Bahig H, Aristophanous M (2018). Prospective in silico study of the feasibility and dosimetric advantages of MRI-guided dose adaptation for human papillomavirus positive oropharyngeal cancer patients compared with standard IMRT. Clin Transl Radiat Oncol.

